# A Novel Role for BDNF-TrkB in the Regulation of Chemotherapy Resistance in Head and Neck Squamous Cell Carcinoma

**DOI:** 10.1371/journal.pone.0030246

**Published:** 2012-01-20

**Authors:** Junegoo Lee, Tilahun Jiffar, Michael E. Kupferman

**Affiliations:** Department of Head and Neck Surgery, MD Anderson Cancer Center, University of Texas, Houston, Texas, United States of America; University of Nebraska Medical Center, United States of America

## Abstract

Mechanisms of resistance for HNSCC to cisplatin (CDDP), the foundational chemotherapeutic agent in the treatment of this disease, remain poorly understood. We previously demonstrated that cisplatin resistance (CR) can be overcome by targeting Trk receptor. In the current study, we explored the potential mechanistic role of the BDNF-TrkB signaling system in the development of CDDP resistance in HNSCC. Utilizing an *in vitro* system of acquired CR, we confirmed a substantial up-regulation of both BDNF and TrkB at the protein and mRNA levels in CR cells, suggesting an autocrine pathway dysregulation in this system. Exogenous BDNF stimulation led to an enhanced expression of the drug-resistance and anti-apoptotic proteins MDR1 and XiAP, respectively, in a dose-dependently manner, demonstrating a key role for BDNF-TrkB signaling in modulating the response to cytotoxic agents. In addition, modulation of TrkB expression induced an enhanced sensitivity of cells to CDDP in HNSCC. Moreover, genetic suppression of TrkB resulted in changes in expression of Bim, XiAP, and MDR1 contributing to HNSCC survival. To elucidate intracellular signaling pathways responsible for mechanisms underlying BDNF/TrkB induced CDDP-resistance, we analyzed expression levels of these molecules following inhibition of Akt. Inhibition of Akt eliminated BDNF effect on MDR1 and Bim expression in OSC-19P cells as well as modulated expressions of MDR1, Bim, and XiAP in OSC-19CR cells. These results suggest BDNF/TrkB system plays critical roles in CDDP-resistance development by utilizing Akt-dependent signaling pathways.

## Introduction

As the sixth most common cancer worldwide, head and neck squamous cell carcinoma (HNSCC) is responsible for 5% percent of all cancers diagnosed in the United States [1]. The evolution of treatment for this disease has progressed to a greater emphasis on the use of cisplatin (CDDP)-based chemotherapy in the neoadjuvant and adjuvant settings. However, the clinical response to CDDP is associated with only a 10–15% improvement in local-regional control of disease, and marginal improvement in overall survival [2], [3], [4]. These sobering results suggest that resistance to CDDP (CR) is a fundamental biological property of this disease. Moreover, tumor recurrences or distant metastasis rarely respond to CDDP-based regimens. Therefore, understanding the mechanisms underlying CDDP resistance may provide strategic targets and promising treatments against HNSCC.

Platinum-based chemotherapeutic agents, which include CDDP and carboplatinum, function therapeutically primarily by inducing DNA damage in rapidly dividing cells, thereby inducing apoptosis and cell death. Various mechanisms have been reported to contribute to the development of resistance against platinum-based chemotherapeutic agents including: (1) increased drug efflux; (2) decreased drug influx; (3) enhanced detoxification through GST-π activation; (4) suppressed apoptosis; and (5) enhanced DNA repair mechanisms [5], [6]. Among these mechanisms, the Tyrosine Receptor Kinase B and Brain Derived neurotropic Factor (TrkB/BDNF) system has been reported to be responsible for chemoresistance, through Akt/PI-3K and MAPK signaling pathways, in selected models of cancer [7], [8]. However, TrkB-mediated resistance to CDDP has not been studied in HNSCC. Although it is clearly evident that TrkB/BDNF system plays a role in tumor progression and metastasis, the molecular mechanisms underlying BDNF-induced chemoresistance development are not fully understood.

The tropomyosin-related kinase B receptor (TrkB) belongs to receptor tyrosine kinase family and triggers its intracellular signals with its ligands, brain derived neurotrophic factor (BDNF) and neurotrophin 4 (NT4). TrkB has been shown to be over-expressed in various cancer types and is associated with poor prognosis [9], [10]. Cumulative evidence indicates TrkB is responsible for tumor progression such as invasion, metastasis, angiogenesis, and resistance against therapeutic agents [11], [12], [13]. Mechanistically, the activation of TrkB by BDNF has shown to activate various intracellular signaling pathways including Akt, Src, or MAPK resulting in cell proliferation, and apoptosis resistance in models of human cancer [14], [15]. While our previous studies have shown that the resistance of HNSCC to CDDP-based therapy can be overcome with inhibition of TrkB pathways, the mechanisms for this phenomenon has yet to be elucidated [13].

In previous studies, we reported that both TrkB and BDNF were overexpressed in HNSCC, and that modulation of TrkB expression altered the invasive phenotype in this disease [9]. Moreover, we identified TrkB as a potential target for therapy in CDDP-resistant HNSCC [13]. These findings led us to investigate the mechanisms underlying CDDP-resistance induced through BDNF\TrkB in HNSCC. In the current study, we demonstrate a novel molecular mechanism of action for TrkB/BDNF in modulating the response of HNSCC to cisplatin-based treatment, primarily through differential apoptotic response. These results provide a potential strategic approach for reversing cisplatin resistance in aggressive HNSCC.

## Materials and Methods

### Cell lines and Reagents

The head and neck squamous carcinoma cell (HNSCC) lines OSC-19 [9], [13], [16] , HN-5 [9], [13], and UMSCC1 [13] cells were maintained with DMEM supplemented with 2 mM L-glutamine, 0.1 mM MEM non-essential amino acids, 1 mM sodium pyruvate, MEM vitamin solution 100 U/ml streptomycin, penicillin, vitamin and 10% FBS as described previously. OSC-19 and HN-5 cell lines were treated with low dose of cisplatin for six months to generate a cisplatin resistant strains of both cell lines. For end-point PCR analysis, PCR primer set for GAPDH (VHPS-3541) was purchased from Real Time Primers (Elkins Park, PA, USA). Three AKT siRNA oligo pairs (5′ ‘CUCACAGCCCUGAAGUACU 3′, 5′ AGUACUUCAGGGCUGUGAG 3′; 5′ GAGACUGACACCAGGUAUU 3′, 5′ AAUACCUGGUGUCAGUCUC 3′; 5′ GUGCCAUGAUCUGUAUUUA 3′, 5′ UAAAUACAGAUCAUGGCAC 3′) and a non-target universal negative control (5′ GCGCGAUAGCGCGAAUAUATT 3′, 5′ UAUAUUCGCGCUAUCGCGCTT 3′) were purchased from Sigma-Aldrich (Saint Louis, MO) to knock down AKT. A pool of the three AKT siRNA oligos was used to knock down AKT. The following antibodies were used for western blot analysis: goat anti-rabbit antibody (Santa Cruz, sc-2004), goat ant-mouse antibody (sc- 2005); p-Akt(4058), Akt1(2938), p-Src(2101), Src(2108), p-MAPK(9101), MAPK(9102), Bim(2819), GST-π(3369), PARP(9532), Cleaved PARP(9541), and BAX were from Cell Signaling (Danvers, MA); TrkB(sc-8316), GSK-3b(sc-9166), and MDR1(sc-55510) were from Santa Cruz Biotechnology, Inc. (Santa Cruz, CA); and GAPDH (AM4300, Ambion, Austin, TX). Cisplatin and MTT purchased from Sigma (St. Louis, Mo). An Akt inhibitor, triciribine (TCN) was obtained from Berry Associates (Dexter, MI).

### Western blotting (WB)

All western blotting analysis was performed as described previously [17]. Briefly, five to fifty microgram of total protein was loaded to 8 to 12% SDS-PAGE, and transferred onto PDVF membrane. The membrane was blocked with 5% milk in TBST and incubated with the appropriate primary and secondary antibodies and developed with Lumi-Light Western Blotting detection kit (Roche Applied Sciences, Indianapolis, IN). Densitometry data were analyzed by using Un-Scan-It gel Software (Silk Scientific Corp., Orem, UT) and statistical analysis was performed by utilizing either conventional Student's *t* test or ANOVA followed by *post hoc* comparisons based upon modified Newman-Keuls-Student procedure, where appropriate. Results are reported as mean +/− SEM. A *p* value of <0.05 was considered significant and all are paired and two-tailed.

### Plasmid Transfections

Short-hairpin RNA (shRNA) constructs targeting TrkB (Cat. TR320436, *Origene*, Rockville, MD) were introduced into cells via retroviral infection or with lipofectamine2000 reagent according to the manufacturer's protocol, as previously described [16]. For TrkB over-expression, pcDNA3.1 plasmids containing the full length cDNA sequences for human TrkB was stably transfected into UMSCC1 cells and selected with G418. TrkB expression was confirmed by SDS-PAGE gel analysis and end-point RT-PCR analysis.

### RT-PCR

Extraction of total RNAs was conducted by using the Qiagen RNeasy Mini kit (Valencia, CA). The quality and quantity of extracted RNA was analyzed by using Nanodrop (ND-1000) spectrophotometer (NanoDrop Technologies, Inc., Wilmington, DE) at the absorbance ratio of 260 and 280 nm. About 700 ng RNA was used as the template for semi-quantitative RT-PCR analysis to determine expression of TrkB at mRNA level. To determine expression level of TrkB at HNSCC, we conducted one-step RT-PCR reaction with SuperScript® III One-Step RT-PCR System with Platinum®Taq (12574-026, Invitrogen, Carlsbad, CA) with the Gene Amp PCR System 9700 (AB Applied Biosystems, Carlsbad, CA), according to manufacturer's instruction. For RT-PCR, the primers used were human TrkB 5′ CCC ACT CAC ATG AAC AAT GG 3′ (forward) and 5′ TCA GTG ACG TCT GTG GAA GG 3′ (reverse); human BDNF 5′ AAA CAT CCG AGG ACA AGG TG 3″ (forward) and 5′ AGA AGA GGA GGC TCC AAA GG 3′ (reverse); GAPDH (Real Time Primers, Elkins Park, PA), 5′ GAG TCA ACG GAT TTG GTC GT 3′ (forward) and 5′ TTG ATT TTG GAG GGA TCT CG 3′ (reverse). After a reverse transcription reactions at 55°C for 30 minutes, there was an initial 10-minute denaturation at 95°C followed by the 40 cycles run consists of a 15-second denaturation step at 94°C and an annealing step at 60°C for 30 seconds, and extension step at 68°C for 1 minute. The density of bands was determined by using Un-Scan-It gel Software (Silk Scientific Corp., Orem, UT) and statistical analysis was performed by utilizing either conventional Student's *t* test. The PCR results were averaged with three independent experiments, which were used in the statistical analysis. Results were normalized to GAPDH.

### Cell Proliferation Assay with MTT

To calculate IC50 of CDDP in various HNSCCs, MTT assay was conducted. Fifteen hundred cells were seeded into each well of 96-well plates and cultured overnight. Cells were treated with defined concentrations of CDDP for three days, followed by the MTT assay. Briefly, 25 ul of 5 mg/ml MTT was added to each well. After three hour incubation with MTT, the media was removed carefully. The formed dye was dissolved with 100 ul DMSO and the absorbance of each well was read using a micro-plate reader. Six or eight samples were treated with same concentration of CDDP. Experiments were repeated at least three times. CDDP IC_50_ was calculated using the GraphPad Prism Software (GraphPad Software, Inc.).

### ELISA Assay

To determine expression level of BDNF, we performed ELISA assay with BDNF E_max_® ImmunoAssay System (G7610, Promega, Madison, WI) based on the manufacturer's instructions. Briefly, cells were cultured to 80% confluence followed by replacement of fresh culture media grown for 48 hours before collecting the medium. This medium was centrifuged at 1000 g for 10 minutes and used for determining BDNF concentration.

### Immunohistochemistry

Tissues for immunochemistry were obtained from mice as previously reported [16]. Immunohistochemistry was conducted as previously described [17], using anti-XiAP (1∶100, catalogue # IMG-5770; IMGENEX, San Diego CA, USA), anti-Ki67 (1∶50, Dako, Carpinteria, CA, Catalogue # M7240), or anti-Bim antibody (1∶100, catalogue # 2933; Cell Signaling, Danvers, MA, USA).

Quantification of immuohistochemistry in mouse tumor xenografts was done by counting positive cells in high power field (200×) using Image-Pro MC 6.1 (Media Cybernetics, Inc., Bethesda, MD). Values were calculated from sections of at least 4 different tumors.

### STR profile nalysis of parental and CDDP-Resistant cell lines

Extraction of genomic DBA (gDNA) was conducted by using Puregene Genomic DNA purification Kit (Gentra Systems, MN, Minnesota) following instructions provided by the manufacturer. The amount of extracted DNA was analyzed by using Nanodrop (ND-1000) spectrophotometer (NanoDrop Technologies, Inc., Wilmington, DE). STR DNA fingerprinting analysis to determine isogenecity between parental cells and chemoresistant cells at the Characterized Cell Line Core (CCSG) of the MD Anderson Cancer Center. Cell lines were validated by STR DNA fingerprinting using the AmpFℓSTR Identifiler kit according to manufacturer instructions (Applied Biosystems cat 4322288). The STR profiles were compared to known ATCC fingerprints (ATCC.org), to the Cell Line Integrated Molecular Authentication database (CLIMA) version 0.1.200808 (http://bioinformatics.istge.it/clima/) (Nucleic Acids Research 37:D925-D932 PMCID: PMC2686526) and to the MD Anderson fingerprint database.

## Results

### TrkB is up-regulated in cisplatin-resistant head and neck squamous carcinoma cell lines

**Figure 1:**
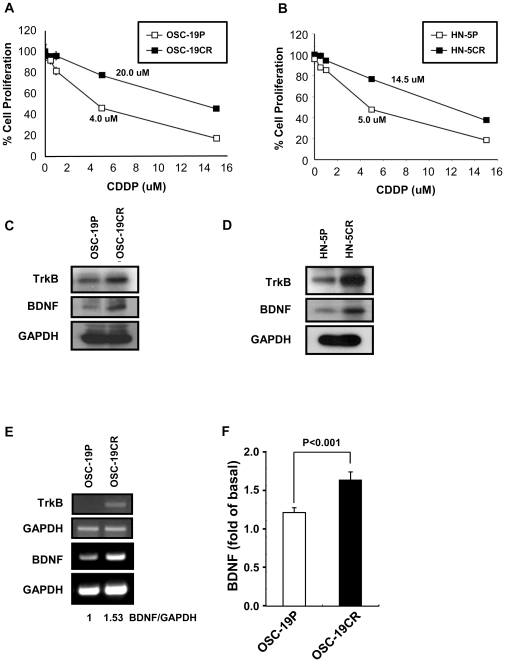
CDDP-resistant HNSCC showed up-regulation of TrkB and BDNF expression. **A and B**, IC50 values for parental cells and its CDDP-resistant cells, OSC-19, A, and HN-5, B. Cell viability was measured by MTT assay 3-day after incubation with CDDP. Each point shows mean ± SDE. IC50 values were calculated with the prism software form the GraphPad Software. **C and D**, Lysates from parental cells and its CDDP-resistance cells were analyzed by western blot assay. After lysates were separated with 12% (BDNF) and 8% (TrkB) SDS-PAGE gels membrane was exposed to proper antibodies. **E**. The expression levels of TrkB and BDNF were determined with OSC-19 parental (OSC-19P) and its CDDP-resistance cells (OSC-19CR). Total RNA was extracted from both OSC-19P and OSC-19CR cells and RT-PCR was performed as described at materials and methods. Cloned products were separated with 1% Agarose gel. **F**. After 24 hours cell culture, medium was collected and the concentration of BDNF was determined with ELISA kit.

We examined expression of TrkB and BDNF levels in cisplatin-resistant head and neck squamous cell carcinoma cell lines (OSC-19CR and HN-5CR, respectively), as previously described [13]. These CR cells were confirmed for their isogenic lineage by short tandem repeat genotyping (STR, Table S1). The degree of CDDP resistance was determined with the MTT assay, and long-term exposure to CDDP induced a 3–4 fold increase in the IC_50_ of each cell line. (Figure 1A & 1B). We next established that both TrkB and BDNF expression was up-regulated in OSC-19CR and HN-5CR cells, compared to the parental cell lines. These findings were confirmed by ELISA and RT-PCR for BDNF and by western blot and RT-PCR for the TrkB receptor (Figure 1C, 1D, 1E & F). These results suggested that the chemoresistant phenotype were associated with altered expression of BDNF-TrkB and confirmed our prior results [9], [13], [16].

### CDDP-resistant cells up-regulate an anti-apoptotic expression profile

**Figure 2:**
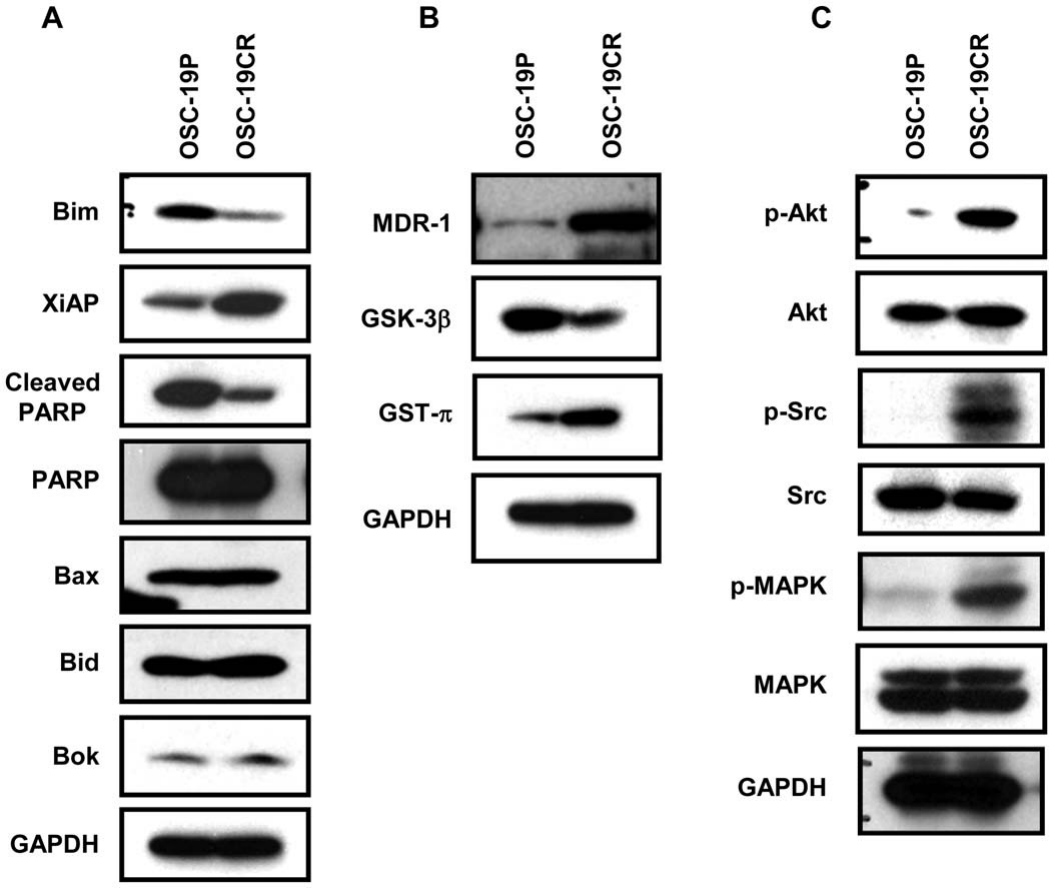
CDDP resistant cells modulate expression of molecules related with cell survival. **A**. Apoptotic molecules, **B**. Drug resistance-related molecules, and **C**. intracellular signal molecules.

As our previous studies supported the notion that downstream dysregulation in drug metabolism and enhanced apoptosis resistance were associated with TrkB/BDNF signaling, we next hypothesized that the observed CR phenotype was induced by alterations in apoptotic pathways. Substantial upregulation of anti-apoptotic proteins and suppression of pro-apoptotic proteins were seen in the CR cell lines. In CDDP-resistant cells, XiAP, an inhibitor of apoptosis, was up-regulated, while Bim, a pro-apoptotic maker, was down-regulated. However, we failed to observe any alteration of other apoptotic markers including Bax, Bid, Bok and a slight down regulation in the basal expression of cleaved PARP in the resistant cell lines (Figure 2A). In order to understand the complete picture of the expression of the BCL2 family of proteins in regulation of Cisplatin resistance, we analyzed the expression of anti-apoptotic molecules of the BCL2 family and showed that BCL-XL and Survivin were upregulated in the CDDP-resistant cell lines compared to their parental counterparts (Figure S1A).

Among the enzymes associated with drug resistance, increased expression of multidrug resistance 1 (MDR1), and glutathion S tranferase-π (GST-π) was noted (Fig. 2B), which confirmed our previous findings vis-à-vis the role of KiSS1 in mediating CR behavior, and is consistent with previous findings of GSK3β-mediated TrkB-induced cell protection from chemotherapy [16], [18]. To determine whether downstream signaling in known pathways of apoptosis resistance was associated with these findings, we next compared the activation of intracellular cascades in the parental and CR cells. While MAPK phosphorylation was consistently elevated among the resistant cell lines, increased Src and Akt phosphorylation was also observed, suggesting that these selected pathways coordinated CDDP metabolism and the resulting inhibition of cell death in the presence of CDDP (Fig. 2C).

### CDDP induces differential signaling in CDDP resistance

**Figure 3:**
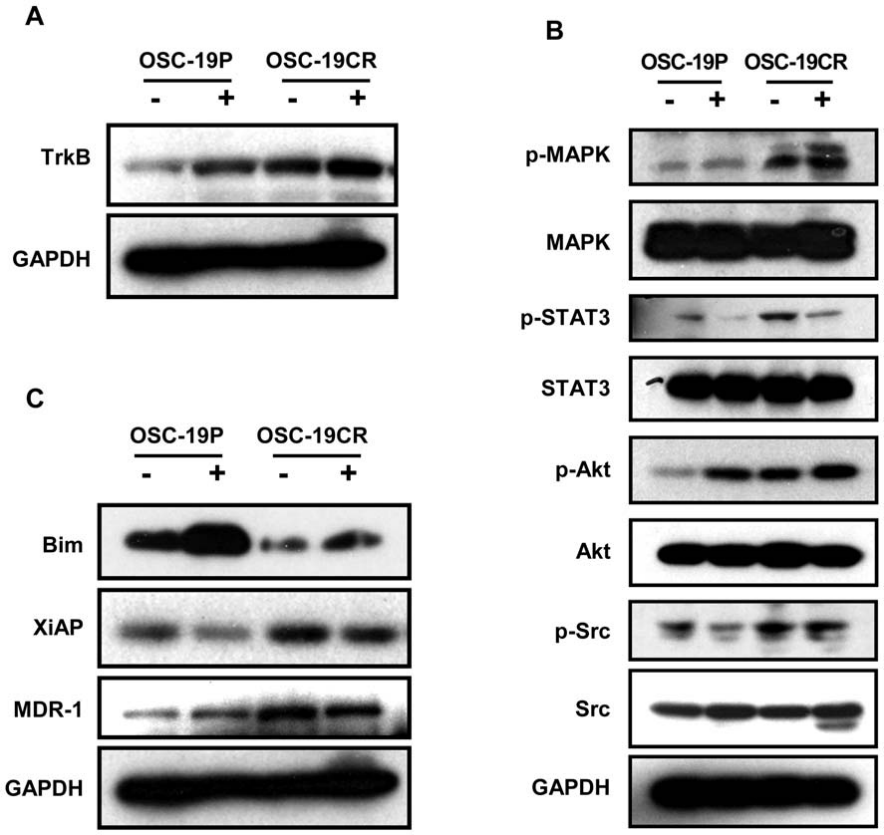
Differential response of OSC-19P and OSC-19CR to CDDP. Cell lysate was collected following 5 uM of CDDP and separated with SDS-PAGE gel. Membrane was then incubated with indicated antibodies. **A**. Expression of TrkB was determined in both OSC-19P and OSC-19CR following 5 uM of CDDP treatment. **B**. Phosphorylation of Akt, Src, MAPK, and STAT-3 was determined following 5 uM CDDP treatment. Levels of phosphorylated kinases were normalized with expression of total kinases. **C**. 5 uM of CDDP modulates expression of Bim, XiAP, and MDR1. Expression levels of Bim, XiAP, and MDR1 were determined by Western blot analysis.

As our previous data supported the concept that activation of anti-apoptotic proteins was partially driven by BDNF in drug-sensitive cells, we explored whether this phenomenon was differentially altered in the drug-resistant cell lines. First, to examine the impact of CDDP treatment on TrkB expression, we compared the parental cells to the resistant cells for receptor expression. Western blot analysis revealed that after 24 hours of exposure to CDDP, a substantial increase in receptor expression was seen in the sensitive cells (125% increase), but a blunted response was noted in the CR cells due to elevated basal levels (25% increase) (Figure 3A). However, we observed an induction of pro-survival pathways in the CR cells, as evidenced by phosphorylation of MAPK (50% increase) and Akt (30% increase), while baseline Src activation remained elevated (Figure 3B). The results were consistent with our previous findings when comparing the basal activity of anti-apoptotic pathways in the parental and resistant lines. Additionally, CDDP induced genes favoring cell death in the parental cells, particularly the pro-apoptotic molecule, Bim. Treatment of CDDP resistant cell line with a relatively high dose of CDDP for 24 hrs did not alter the basal expression of XIAP and MDR1 suggesting a stable phenotypic change of these molecules in response to drug treatment (Figure 3C). It is interesting to note that, overall, CDDP-resistant cell lines have an elevated level of STAT3 phosphorylation, but no change is observed when cells are treated with CDDP suggesting that STAT3 may not be playing a significant role in the development of resistance in Head and Neck cell lines (Fig. 3B). These results demonstrate that CDDP resistant cell lines harbor a differential molecular response profile that may be due in part to enhanced pro-survival signaling and constitutive apoptotic resistance.

### BDNF regulates signaling and apoptosis in drug-sensitive HNSCC

**Figure 4:**
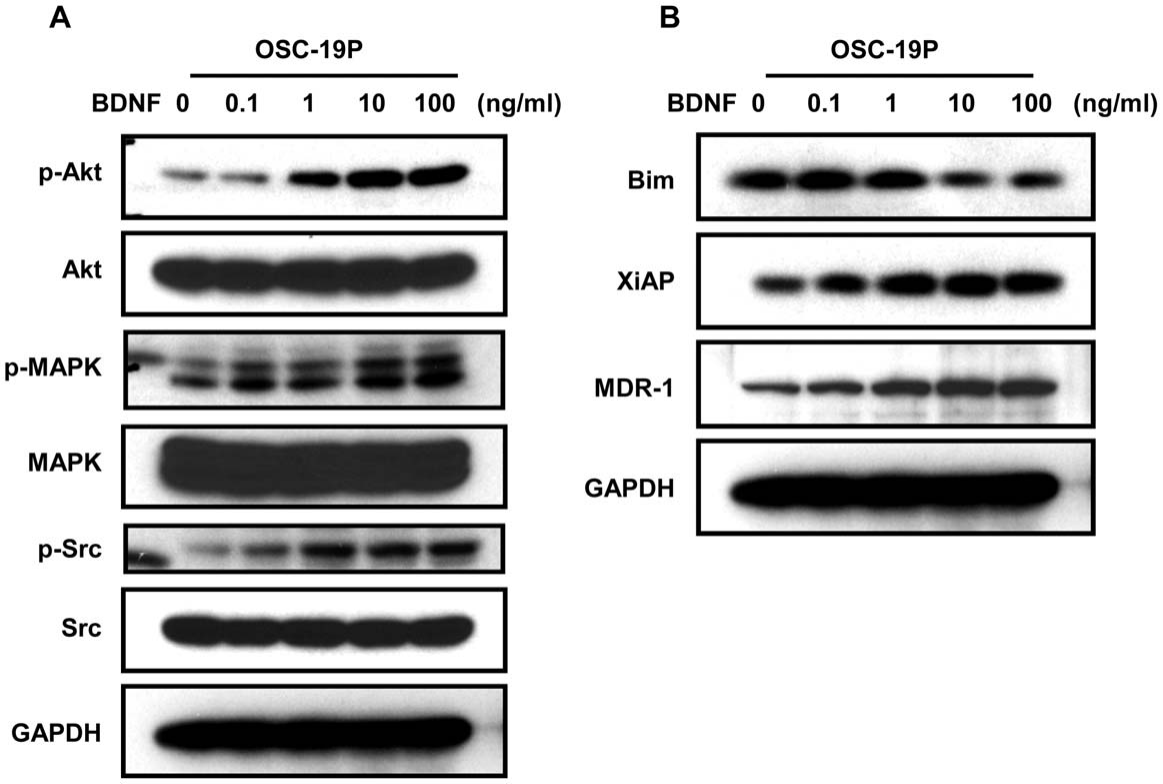
BDNF induces signals and modulates MDR1, Bim, and XiAP in CDDP sensitive cells. **A**. Dose-dependent up-regulation of intracellular signals, Akt, Src, and MAPK in OSC-19P. The culture reaches ∼80% confluent, cells were starved for overnight with medium without serum. Lysate was collect after 24 hour treatment with 100 ng/ml BDNF and separated with SDS-PAGE gel and membrane was incubated with indicated antibodies. **B**. Western blot analysis of XiAP, Bim, and MDR1 following treatment with various concentration of BDNF for 24 hours (0.1, 1, 10, and 100 ng/ml, respectively).

We next sought to determine whether exogenous BDNF could induce the molecular alterations that were consistent with those seen in the CDDP-resistant cells. To determine whether TrkB/BDNF signaling impacted the induction of apoptotic pathways in CDDP-sensitive cell lines, we analyzed the alterations in the phosphorylation status of various signaling molecules. After exposure to BDNF for 24 hours, stimulation with BDNF resulted in increased phosphorylation of various kinases, Akt, Src, and MAPK in parental cells, consistent with previous studies regarding its role in tumor proliferation and apoptosis (Figure 4A) [13]. The activation of these kinases was noted in a dose-dependent manner, even at low BDNF concentrations. We also measured expression levels of XiAP, Bim, and MDR1, which were previously found to be dysregulated in our system. In these experiments, we found that level of XiAP, an inhibitor of apoptosis, was induced by a 5.3-fold change under the influence of BDNF exposure (Figure 4B). In contrast, the expression of the pro-apoptotic protein Bim was reduced by 70% in a dose-dependent manner after exposure to BDNF (Figure 4B). In line with these findings, the expression of MDR1, the cisplatin-efflux complex was enhanced by 60% when cells were exposed to BDNF (Figure 4B). These results suggests that exposure to exogenous BDNF could contribute to a molecular profile that mimicked the CDDP-resistant one.

### Alterations in CDDP-response is mediated by TrkB

**Figure 5:**
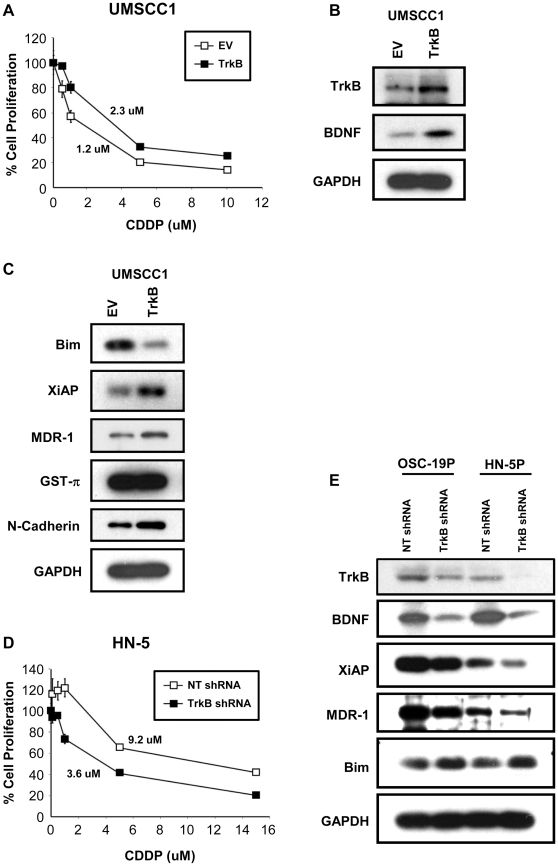
TrkB altered sensitivity to CDDP in HNSCC. **A**. UMSCC1 cells were transfected with a vector containing wild-type TrkB (TrkB) or an empty vector (EV). Cells were then exposed to CDDP for addressing increasing resistance of TrkB over-expressed UMSCC1 to CDDP. MTT cell viability assay was conducted to determine the IC50 values. **B.** expression efficacy was confirmed by Western Blot analysis. BDNF expression level was also determined. **C.** Expression levels of XiAP, Bim, MDR1, GST-p, and N-cadherin were determined with over-expressed UMSCC1 cells. Both OSC-19 and HN-5 cells were stably transfected with a short-hairpin RNA construct targeting TrkB (TrkB shRNA) or a non-targeting, short-hairpin RNA construct (NT shRNA). **D.** Both HN-5P and HN-5CR cells were then treated with various concentration of CDDP and analyzed to address the effect of TrkB down-regulation on sensitivity to CDDP by suppressing TrkB expression. **E.** Down-regulation of TrkB expression was confirmed by Western blot analysis. Cell lysates of suppressing TrkB were separated with SDS-PAGE and membrane was incubated with BDNF, Bim, XiAP, and MDR1 antibodies. Expression levels of TrkB, BDNF, Bim, XiAP, and MDR1 were normalized with GAPDH.

Although the previous results highlighted an association between CDDP resistance and enhanced TrkB signaling, we next sought to test whether this relationship was directly mediated by receptor activity. Initially, we over-expressed TrkB in the low-expressing UMSCC1 cell line and compared the response of these genetically-altered cells to CDDP. A moderate induction of CDDP resistance was noted (Figure 5A & 5B) in cells with genetically upregulated TrkB, compared to those transfected with an empty vector, confirming our prior results [13]. As shown in Figure 5A, over-expression of TrkB increased in the IC_50_ from 1.2 uM (empty vector) to 2.3 uM significantly (p<0.05). Corresponding to this altered phenotype, we observed that Bim was down-regulated, suggesting that the CDDP resistance may have been due in part to suppressed apoptosis through a TrkB-directed mechanism (Figure 5C). Additionally, we found that the expression of MDR1 and XiAP were induced by genetic upregulation of TrkB, a finding that was in line with our results from exogenous BDNF exposure. To further establish this direct link, we genetically suppressed the expression of TrkB HNSCC cell lines with high levels of endogenous TrkB, HN-5 and OSC-19. In contrast to the previous experiments, the relative loss of TrkB expression initiated a more cisplatin sensitive response in HNSCC cells, with a nearly 66% reduction in IC_50_ value in the TrkB-knockdowns (Figures 5D and S1B). Additionally, when shRNA-based knockdown of TrkB was induced, an inverse expression change between Bim and MDR1 was seen, which was complementary to the TrkB overexpression results. Corresponding analysis by Western blotting of MDR1 expression revealed attenuated expression, and a reciprocal enhancement in Bim expression (Figure 5E). These findings correlated not only with the previous TrkB inhibition experiments providing greater evidence for a direct role of BDNF-TrkB in modulating therapy resistance in HNSCC. An interesting observation was that knock down of TrkB led to reduced BDNF expression, suggesting a possible autocrine pathway for BDNF/TrkB in in the development of CDDP-resistance in HNSCC. In addition inhibition of TRKB in CDDP-resistant cell line increased sensitivity to CDDP treatment as compared to parental CDDP-resistant cell line (Figure S1C).

### Akt plays a crucial role in TrkB-induced CDDP-resistance development

**Figure 6:**
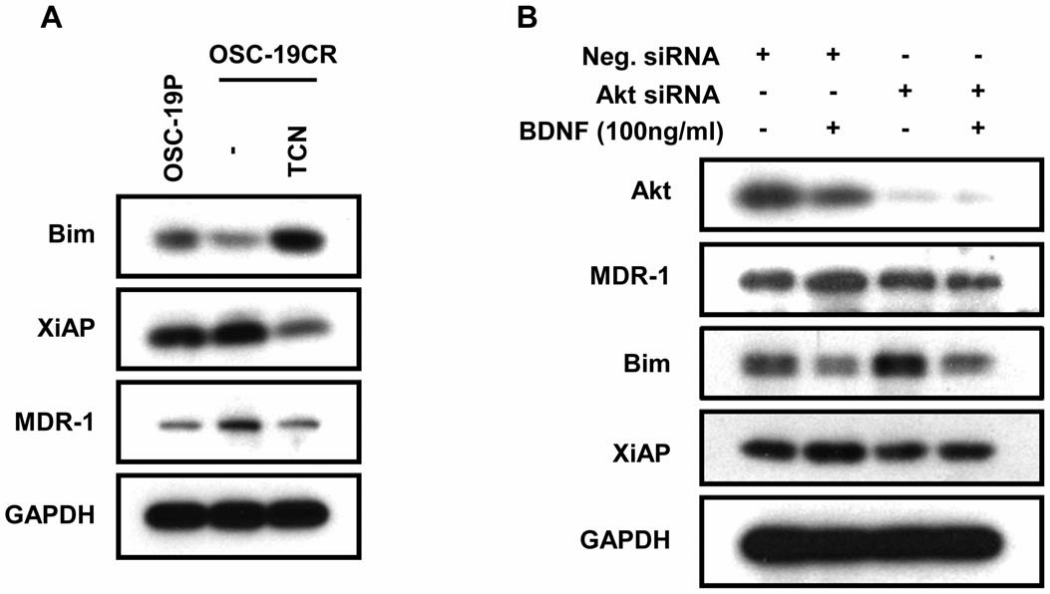
Akt plays a critical role in TrkB-induced CDDP-resistance development. **A**. OSC-19CR cell lysate following treatment with 5 uM of TCN, an Akt inhibitor, was collected and separated with SDS-PAGE to determine expression levels of Bim, XiAP, and MDR1. **B**. Expression levels of Bim, XiAP, and MDR1 were determined with cell lysate following transfected with siRNA against role of Akt in CDDP-resistance development in HNSCC.

We next explored the intracellular signaling pathways that were responsible for mediating TrkB-induced CDDP resistance development in our system. We exposed CR cells to an inhibitor of Akt signaling and assayed for the expression of Bim, XiAP and MDR1 (Figure 6). Selective Akt pathway abrogation led to specific induction of Bim expression, and a significant decrease in MDR1 and XiAP protein expression, as determined by WB analysis. The changes in expression levels of MDR1, XiAP, and Bim in OSC-19CR cells reverted to the levels noted in the parental cells (Figure 6A). We further substantiated these findings with genetic suppression of Akt prior to BDNF exposure. While a non-targeted construct did not alter the expected changes in Bim, MDR1 and XiAP expression, siRNA targeting Akt selectively blocked the BDNF-regulated alteration in apoptosis and drug resistance regulation (Figure 6B). Taken together, these results strongly support our hypothesis that up-regulation of TrkB/BDNF system contributes to CDDP-resistance development through modulating molecules, such as MDR1 and XiAP through Akt-dependent pathway facilitating cell survival against CDDP in HNSCCs.

### Expression of Bim and Ki-67 in animal models of HNSCC

**Figure 7:**
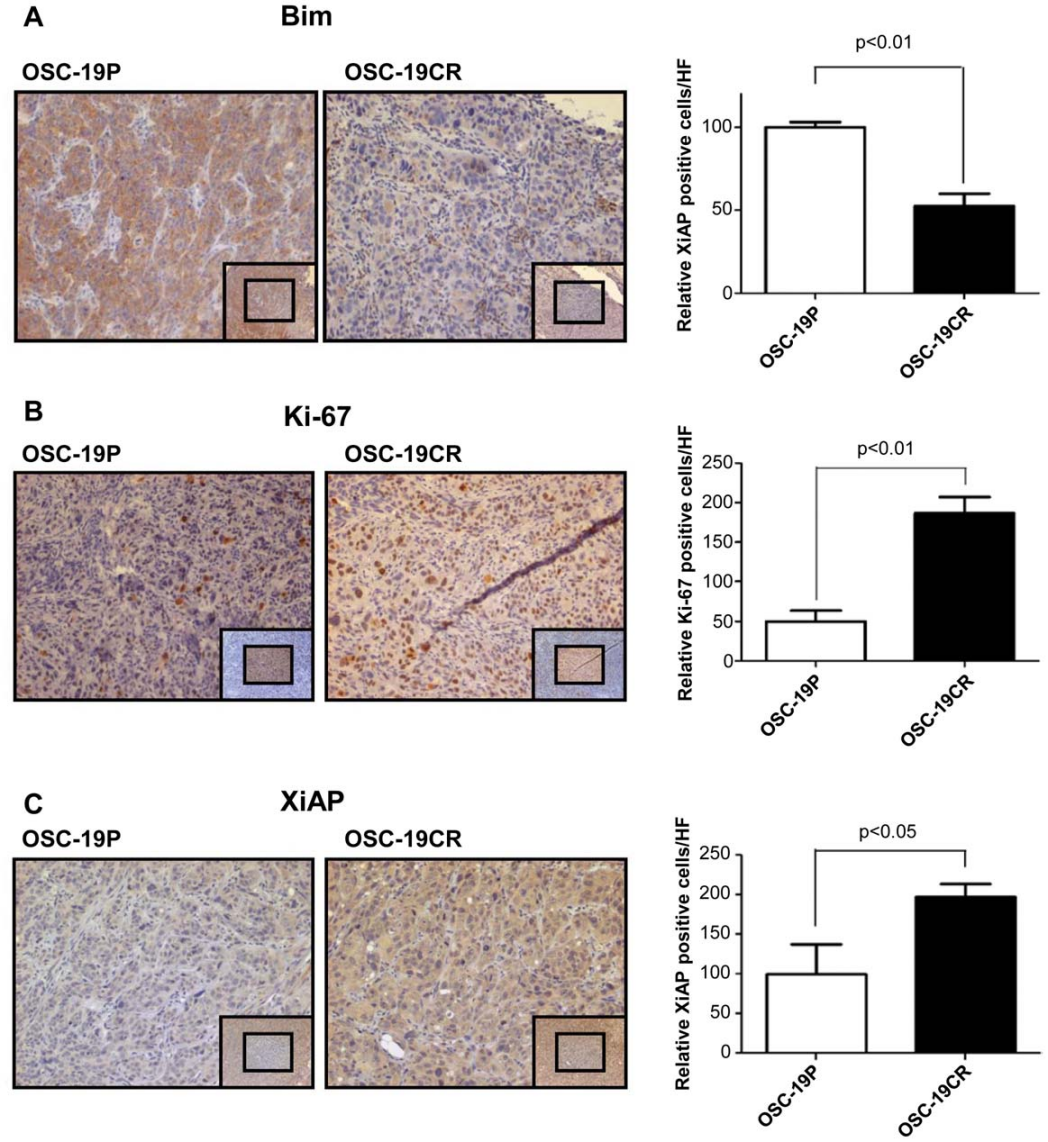
Immunohistochemistry staining of mouse tumor tissue originated from OSC-19P and OSC-19CR. A. Immunohistochemistry staining of OSC-19P (left) and OSC-19CR (right) with Bim. B. IHC staining of OSC-19P (left) and OSC-19CR (right) with Ki-67. The pictures were taken at 100× magnification, the inlet at 50× magnification. (C) IHC staining of OSC-19P (left) and OSC-19CR (right) with XiAP.

To determine whether our in vitro findings were relevant in animal models of HNSCC, we analyzed the expression level of Bim, XiAP, MDR1, and Ki-67 *in vivo* (Figure 7A–C). Mouse-borne tumors derived from parental or CR cell lines were assayed by immunohistochemistry [17]. We confirmed that Bim expression was down-regulated in CDDP-resistant OSC-19CR tumors (48%), compared to the parental tumors. In addition, protein expression of XiAP and Ki-67 expression was increased in CDDP-resistant OSC-19CR tumors (98% and 270%, respectively). However, we failed to show significant up-regulation of MDR1 expression. (data not shown). These data corroborated our hypothesis that chemoresistant tumors were associated with biological markers of apoptotic resistance and enhanced proliferative capacity [16].

## Discussion

Our previous study has shown that the BDNF-TrkB signaling axis contributes to tumor invasion and progression of HNSCC in xenograft animal models [9]. In continuation of our studies, we explored the role of this neurotrophic signaling axis in the development of chemotherapy resistance in HNSCC. Phenotypic characterization of cisplatin-resistant HNSCC cell lines demonstrated an upregulation of both TrkB and its ligand, brain derived neurotrophic factor (BDNF) with a concomitant increase in cell proliferation and apoptosis. Utilizing over-expression and knockdown experiments, we demonstrated the potential for the BDNF-TrkB as a realistic target in overcoming therapy resistance in HNSCC.

Various mechanisms of drug resistance have been identified across differing cancer cell types. One major mechanism that has been identified is enhanced resistance to the induction of apoptosis in response to chemotherapy. In current study, we observed changes in XiAP and Bim expression in the resistant cell lines, compared to the sensitive parental cell lines. Moreover, these changes were induced with exogenous BDNF treatment in the parental cell lines. This evidence indicates that the modulation of both XiAP and Bim expression is due, in part, to up-regulation of BDNF/TrkB system in the CR system. As an inhibitor of apoptosis, XiAP over-expression is a well-described phenomenon in various types of cancer and appears to protect cells from various insults [19]. This occurs through the inhibition of caspase enzymes, which initiate and execute apoptosis [19]. As a pro-apoptotic molecule, Bim has been demonstrated to induce apoptosis by inhibiting anti-apoptotic molecules such as bcl-2, bcl-xL, or bcl-w [20]. In the current study, we showed that Bim expression was reduced in CDDP-resistant HNSCC and exogenous administration of BDNF resulted in reduced Bim expression, a finding that is consistent with effects seen in various disease models including neuroblastoma model [18], [21]. The increase in BDNF can cause in part of Bim degradation through a MAPK-dependent mechanism [18], [21], although this phenomenon has not been evaluated in our system. These findings suggest that down-regulation of Bim by BDNF might be a potential mechanism underlying CDDP-resistance development in the HNSCC. In contrast to our findings, however, Li et al. (2007) reported that reduction in Bim expression through BDNF failed to block the CDDP-induced cell death in a neuroblastoma model [18]. This indicated that perhaps Bim does not play a central role in drug resistance in neuroblastoma, or alternatively, the acquired resistance in our model mechanistically is driven by a direct BDNF-Bim pathway. Further studies will be necessary to elucidate the molecular basis for de novo versus acquired resistance through BDNF in our system. One interesting result regarding the reduction in Bim expression mediated by BDNF is that Bim has been reported to regulate anoikis, apoptosis induced by detachment, in various cancers [22], [23]. Another confounding issue is distinguishing the anti-apoptotic roles of BDNF in anoikis resistance, as opposed to its import in chemotherapy resistance [24], [25].

Resistance to many common anticancer drugs, particularly platinum-based agents, often occurs due to enhanced expression of MDR1 [26], which encodes a P-glycoprotein, calcium-dependent efflux pump. [27], [28], [29]. MDR1 expression has been reported in various types of chemoresistant tumor phenotypes including neuroblastoma, hepatoblastoma, ovarian cancer, and prostate cancer [30]. Epigenetic changes in methylation of its promoter region has been suggested as a mechanism to modulate MDR1 expression [31]. Although change in mRNA stability can contribute to MDR1 expression [32] epigenetic methylation of promoter for MDR1 gene has also been suggested to be a critical mechanism in MDR1 expression at the transcription level and to be associated with poor prognosis in various cancers [33], [34], [35], [36]. In line with our studies, BDNF was shown to induce MDR1 expression in TrkB-overexpressing neuroblastoma cells, at the transcriptional level [37]. These results suggest a possible mechanism underlying up-regulation of MDR1 by TrkB/BDNF through change in both transcription rate and mRNA stability in CDDP-resistant HNSCC.

It is well known that BDNF utilizes PI-3K/Akt, MAPK, and Src signaling pathways to induce various physiological functions including anti-apoptosis and cell proliferation, angiogenesis, anoikis and metastasis [9], [13], [14], [38]. The results of current study also revealed that activation of TrkB by BDNF was enhanced in the CR cell lines, and led to robust induction of the Akt, MAPK, and Src signaling pathways that exceeded the effects in the parental lines. Because Akt is a central node in BDNF signaling and its downstream effects [21], [39], [40], [41], we focused on the contribution of Akt in the development of CDDP resistance. Although we cannot entirely exclude the relevance of MAPK and Src here, our results do not support major roles for these pathways in acquired chemoresistance. Modulation of XiAP and MDR1 expression by BDNF was blocked by both pharmacological and genetic modulation of Akt, suggesting that Akt is responsible for BDNF-induced apoptotic resistance in HNSCC models. In distinction to this, Akt inhibition failed to modulate the BDNF-regulation of Bim expression, perhaps indicated that a bypass mechanism exists for Bim regulation through TrkB. Previously, BDNF-induced BIM down-regulation was found not to be an Akt-dependent effect, but rather a MAPK-dependent pathway [18], [21]. However, the Akt pathway was reported to regulate Bim expression negatively by modulating Forkhead transcription factor in nerve growth factor depravation [42], [43]. This may explain the observed enhancement in Bim expression under conditions of Akt inhibition in the CR cells lines. Further studies are necessary to distinguish the impact of BDNF on the differential regulation of anti-apoptotic systems in HNSCC as it pertains to the regulation of chemotherapy response. Perhaps tumors that have a more “Akt-like” phenotype would be resistant to CDDP-based therapy, a biological behavior that could be reversed with dual BDNF-Akt inhibition.

In conclusion, our findings reveal novel regulatory mechanisms for chemotherapy resistance directed in part by BDNF-TrkB axis in a model of therapy resistant HNSCC. In the current study, we found: (1) both TrkB and BDNF were up-regulated in CDDP resistant HNSCC cell lines (2) a molecular phenotype consistent with apoptotic resistance was associated with acquired CDDP resistance expression (3) exogenous BDNF can induce a CDDP-resistance in CDDP-sensitive cells (4) modulation of TrkB expression resulted in XiAP, Bim, and MDR1 dysregulation, which contributed to CDDP resistance (5) a phenotype that was driven in part by dysfunction Akt signaling mediates CDDP resistance in HNSCCs. Taken together, we have demonstrated a BDNF-TrkB mediated mechanism that underlies CDDP resistance development in HNSCC. These findings may provide insights into potential molecular-based approaches to re-introduce chemotherapy sensitivity in tumors that have developed acquired resistance in HNSCC.

## Supporting Information

Table S1 **STR profile analysis of parental and chemoresistant cells derived from OSC-19 and HN-5 HNSCCs.**
(DOC)

Figure S1 A, CDDP-resistant HNSCC cells showed up-regulation of Bcl-XL and survivin expression but failed to show modulation of Bcl-2 and PUMA expression. B, Small molecule inhibitor of TrkB, AZ64, attenuated Bcl-XL expression in CDDP-resistant HNSCC cell lines. C, Small molecule inhibitor of TrkB, AZ64, sensitized OSC-19CR to cisplatin.
(TIF)
