# Supplementary material for: A Novel Role for BDNF-TrkB in the Regulation of Chemotherapy Resistance in Head and Neck Squamous Cell Carcinoma
Source: PLoS One. 2012 Jan 20;7(1):e30246. doi: 10.1371/journal.pone.0030246 (PMC3262811; doi:10.1371/journal.pone.0030246)
Supplement: Table S1 — STR profile analysis of parental and chemoresistant cells derived from OSC-19 and HN-5 HNSCCs. (DOC) [file pone.0030246.s001.doc]

**Supplementary Table 1. STR profile analysis of parental and chemoresistant cells derived from OSC-19 and HN-5 HNSCCs.**

| NAME | AMEL | CSF1PO | D13S317 | D3S1358 | D5S818 | TH01 | TPOX | vWA |
| --- | --- | --- | --- | --- | --- | --- | --- | --- |
| HN-5P | X | 11,13 | 11 | 18 | 13 | 9.3 | 8,11 | 15,18,19 |
| HN-5CR | X | 11,13 | 11 | 18 | 13 | 9.3 | 8,11 | 15,18,19 |
| OSC-19P | X,Y | 12 | 12 | 14 | 10,13 | 9 | 11 | 14,18 |
| OSC-19CR | X,Y | 12 | 12 | 14 | 10,13 | 9 | 11 | 14,18 |
