# Supplementary figures and images for: A Novel Role for BDNF-TrkB in the Regulation of Chemotherapy Resistance in Head and Neck Squamous Cell Carcinoma
Source: PLoS One. 2012 Jan 20;7(1):e30246. doi: 10.1371/journal.pone.0030246 (PMC3262811; doi:10.1371/journal.pone.0030246)

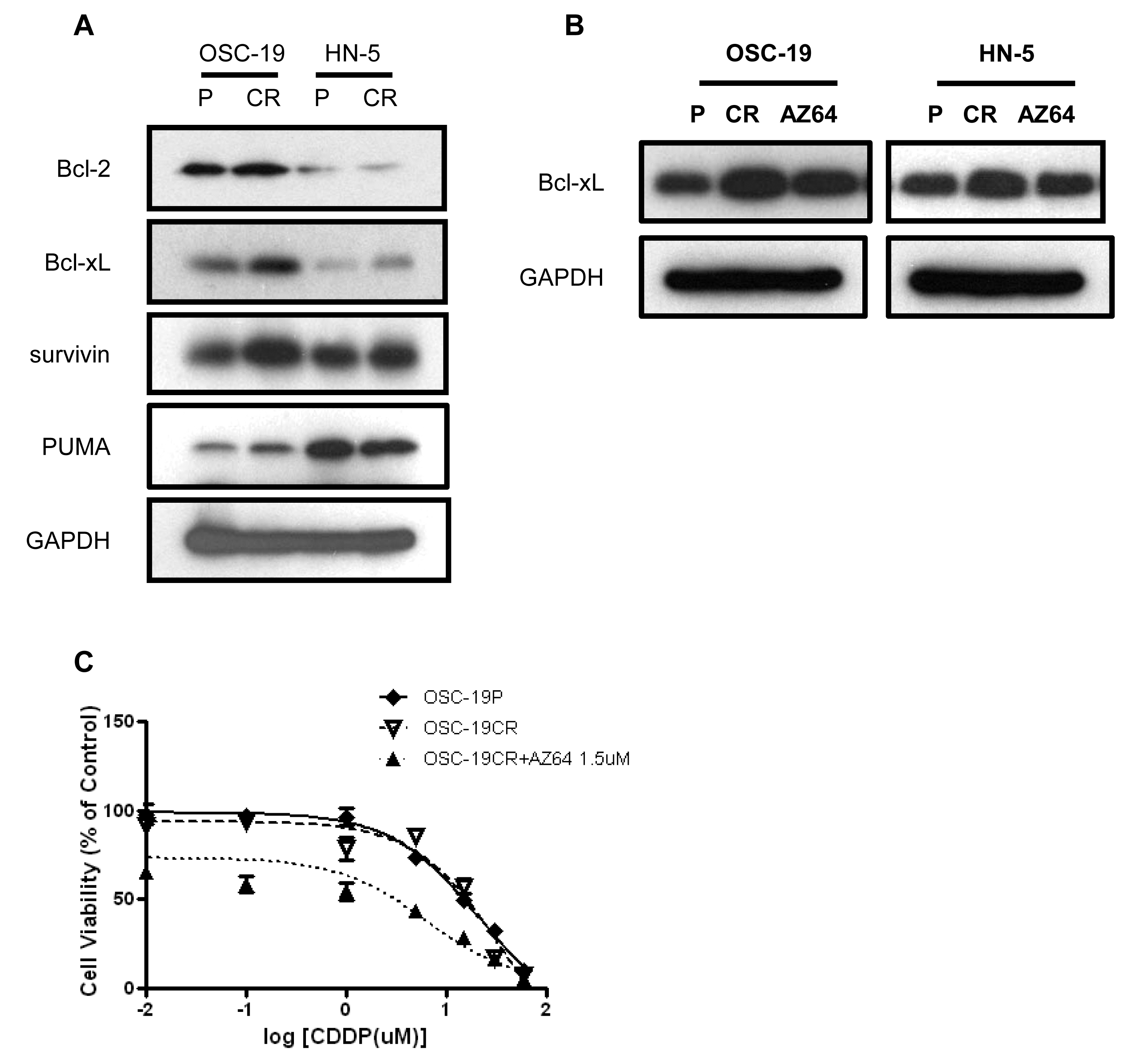

Supplement: Figure S1 — A, CDDP-resistant HNSCC cells showed up-regulation of Bcl-XL and survivin expression but failed to show modulation of Bcl-2 and PUMA expression. B, Small molecule inhibitor of TrkB, AZ64, attenuated Bcl-XL expression in CDDP-resistant HNSCC cell lines. C, Small molecule inhibitor of TrkB, AZ64, sensitized OSC-19CR to cisplatin. (TIF) [file pone.0030246.s002.tif]
